# Supplementary material for: Associations between cesarean delivery and child mortality: A national record linkage longitudinal study of 17.8 million births in Brazil
Source: PLoS Med. 2021 Oct 12;18(10):e1003791. doi: 10.1371/journal.pmed.1003791 (PMC8509988; doi:10.1371/journal.pmed.1003791)
Supplement: S2 Text — Table A: Robson Classification and CD rate. Table B: SMDs before and after matching for the matching covariates, Robson group 1 to 4. Table C: SMDs before and after matching for the matching covariates, Robson group 5. Table D: SMDs before and after matching for the matching covariates, Robson groups 6 to 10. Table E: Logistic regression estimates of the odds of delivery by CD in Robson groups 1 to 4 or by vaginal delivery in Robson group 5 and groups 6 to 10. Table F: Mortality conditional on survival up to 6 days, 27 days, and under 1 year, by mode of delivery in Robson groups 1 to 4 before and after PSM, Brazil 2012 to 2018. Table G: Under-five mortality from external causes of death*, Brazil 2012 to 2018. Table H: HRs from sensitivity analyses for under-five mortality. PSM, propensity score matching; SMD, standardized mean difference. (DOCX) [file pmed.1003791.s002.docx]

**Supporting Material 2**

| **Table A. Robson Classification and caesarean delivery rate** | |  | |  | | |
| --- | --- | --- | --- | --- | --- | --- |
| **Robson Classification** | | **Population size** | | | **CD rate** | |
|  |  | **N** | **%** | | **N** | **%** |
| 1 | Nulliparous women with a single cephalic pregnancy, ≥37 weeks gestation in spontaneous labour | 2,890,039 | 16.21 | | 1,336,141 | 46.23 |
| 2a | Nulliparous women with a single cephalic pregnancy, ≥37 weeks gestation who had labour induced | 1,206,375 | 6.77 | | 310,326 | 25.72 |
| 2b | Nulliparous women with a single cephalic pregnancy, ≥37 weeks gestation who had pre labour CD | 1,674,370 | 9.39 | | 1,674,370 | 100 |
| 3 | Multiparous women without a previous uterine scar, with a single cephalic pregnancy, >37 weeks gestation in spontaneous labour | 3,194,792 | 7.92 | | 618,340 | 19.35 |
| 4a | Multiparous women without a previous CD, with a single cephalic pregnancy, ≥37 weeks gestation who had labour induced | 1,160,758 | 6.51 | | 131,800 | 11.35 |
| 4b | Multiparous women without a previous CD, with a single cephalic pregnancy, ≥37 weeks gestation who had pre labour CD | 680,235 | 3.82 | | 680,235 | 100 |
| 5 | All multiparous women with at least one previous CD, with a single cephalic pregnancy, ≥37 weeks gestation | 4,073,288 | 22.84 | | 3,490,187 | 85.68 |
| 6 | All nulliparous women with a single breech pregnancy | 258,992 | 1.45 | | 234,081 | 90.38 |
| 7 | All multiparous women with a single breech pregnancy including women with previous CD | 371,536 | 2.08 | | 317,616 | 85.49 |
| 8 | All women with multiple pregnancies including women with previous CD | 424,183 | 2.38 | | 352,631 | 83.13 |
| 9 | All women with a single pregnancy with a transverse or oblique lie, including women with previous CD(s) | 41,882 | 0.23 | | 40,564 | 96.85 |
| 10 | All women with a single cephalic pregnancy <37 weeks gestation, including women with previous CD | 1,853,741 | 10.40 | | 933,116 | 50.34 |

CD: caesarean delivery

| **Table B. Standardized mean differences (SMD) before and after matching for the matching covariates, Robson group 1-4.** | | | | | | | |
| --- | --- | --- | --- | --- | --- | --- | --- |
|  |  | **Before matching** | | | **After matching** | | |
|  |  | **Vaginal delivery** | **CD** |  | **Vaginal delivery** | **CD** |  |
|  |  | **N(%)** | **N(%)** | **SMD** | **N(%)** | **N(%)** | **SMD** |
| **Maternal age** | < 14 years | 14,001 (0.2) | 6,443 (0.3) | 0·113 | 5,425 (0.3) | 6,006 (0.3) | 0.036 |
|  | 14-15 years | 191,377 (3.2) | 69,987 (2.9) |  | 64,378 (3.3) | 65,765 (3.3) |  |
|  | 16-17 years | 536,240 (8.9) | 187,550 (7.8) |  | 175,567 (8.9) | 177,185 (9.0) |  |
|  | 18-19 years | 743,865 (12.3) | 260,342 (10.9) |  | 240,426 (12.2) | 244,010 (12.4) |  |
|  | 20-21 years | 770,531 (12.7) | 275,411 (11.5) |  | 245,522 (12.5) | 252,584 (12.8) |  |
|  | 22-23 years | 700,100 (11.6) | 253,850 (10.6) |  | 217,519 (11.0) | 224,727 (11.4) |  |
|  | 24-25 years | 625,770 (10.3) | 240,136 (10.0) |  | 194,640 (9.9) | 199,737 (10.1) |  |
|  | 26-27 years | 561,291 (9.3) | 230,691 (9.6) |  | 179,141 (9.1) | 179,024 (9.1) |  |
|  | 28-29 years | 492,430 (8.1) | 216,641 (9.0) |  | 163,431 (8.3) | 158,557 (8.0) |  |
|  | 30-31 years | 422,886 (7.0) | 196,583 (8.2) |  | 145,251 (7.4) | 136,782 (6.9) |  |
|  | 32-33 years | 338,927 (5.6) | 157,565 (6.6) |  | 1,16,582 (5.9) | 109,056 (5.5) |  |
|  | 34-35 years | 259,155 (4.3) \| | 121,468 (5.1) |  | 89,712 (4.6) | 84,682 (4.3) |  |
|  | 36-37 years | 180,928 (3.0) | 827,23 (3.5) |  | 61,143 (3.1) | 58,898 (3.0) |  |
|  | 38-39 years | 114,168 (1.9) | 51,056 (2.1) |  | 38,188 (1.9) | 37,494 (1.9) |  |
|  | 40-41 years | 62,765 (1.0) | 28,198 (1.2) |  | 20,745 (1.1) | 21,319 (1.1) |  |
|  | 42-43 years | 28,093 (0.5) | 12,367 (0.5) |  | 8,624 (0.4) | 9,601 (0.5) |  |
|  | 44-45 years | 9,385 (0.2) | 4,086 (0.2) |  | 2,669 (0.1) | 3,261 (0.2) |  |
|  | 46-47 years | 2,230 (0.0) | 1,053 (0.0) |  | 624 (0.0) | 817 (0.0) |  |
|  | 48-49 years | 564 (0.0) | 259 (0.0) |  | 131 (0.0) | 186 (0.0) |  |
|  | 50+ years | 553 (0.0) | 198 (0.0) |  | 125 (0.0) | 152 (0.0) |  |
| **Marital status** | Single | 2,966,687 (49.4) | 1,000,571 (42.1) \| | 0.148 | 882,842 (44.8) | 895,157 (45.4) | 0.023 |
|  | Widow | 2,971,778 (49.6) | 1,349,994 (56.8) |  | 1,070,680 (54.4) | 1,055,077 (53.6) \| |  |
|  | Divorced | 9,805 (0.2) | 3,600 (0.2) |  | 2,251 (0.1) | 3,038 (0.2) |  |
|  | Married/union | 48,743 (0.8) | 20,713 (0.9) |  | 14,070 (0.7) | 16,571 (0.8) |  |
| **Maternal education** | none | 48,965 (0.8) | 7,687 (0.3) | 0.404 | 5,562 (0.3) | 7159 (0.4) | 0.015 |
|  | 1-3 years | 224,061 (3.7) | 45,912 (1.9) |  | 38,974 (2.0) | 42,827 (2.2) |  |
|  | 4-7 years | 1,427,180 (23.9) | 351,178 (14.8) |  | 323,069 (16.4) | 33,025 (16.8) |  |
|  | 8-12 years | 3,772,446 (63.1) | 1,478,172 (62.5) |  | 1,289,022 (65.4) | 1,328,872 (67.5) |  |
|  | 12+ years | 507,375 (8.5) | 484,385 (20.5) |  | 313,216 (15.9) | 260,732 (13.2) |  |
| **Maternal ethnicity** | White | 1,739,617 (29.6) | 867617(37.3) | 0.198 | 688,732 (35.0) | 673,024 (34.2) | 0.006 |
|  | Black | 3,86,903 (6.6) | 113892 (4.9) |  | 100,525 (5.1) | 105,163 (5.3) |  |
|  | Asian | 23,993 (0.4) | 8377 (0.4) |  | 6,007 (0.3) | 7,512 (0.4) |  |
|  | Mixed race | 3,651,191 (62.0) | 1329565 (57.1) |  | 1,167,492 (59.3) | 1,175,644 (59.7) |  |
|  | Indigenous | 84,390 (1.4) | 9027 (0.4) |  | 7,087 (0.4) | 8,500 (0.4) |  |
| **Year of birth** | 2012 | 779,422 (12.9) | 313,860 (13.1) | 0.030 | 252,170 (12.8) | 253,398 (12.9) | 0.004 |
|  | 2013 | 805,525 (13.3) | 332,498 (13.9) |  | 264,566 (13.4) | 266,583 (13.5) |  |
|  | 2014 | 853,197 (14.1) | 352,029 (14.7) |  | 286,071 (14.5) | 285,863 (14.5) |  |
|  | 2015 | 901,913 (14.9) | 347,777 (14.5) |  | 288,765 (14.7) | 288,038 (14.6) |  |
|  | 2016 | 876,859 (14.5) | 337,594 (14.1) |  | 280,883 (14.3) | 280,749 (14.3) |  |
|  | 2017 | 906,550 (15.0) | 348,366 (14.5) |  | 290,892 (14.8) | 290,798 (14.8) |  |
|  | 2018 | 931,891 (15.4) | 364,483 (15.2) |  | 306,496 (15.6) | 304,414 (15.5) |  |
| **Sex of the newborn** | Female | 3,011,634 (49.7) | 1,131,892 (47.2) | 0.050 | 941,881 (47.8) | 946,956 (48.1) | 0.005 |
|  | Male | 3,043,206 (50.3) | 1,264,449 (52.8) |  | 1,027,962 (52.2) | 1,022,887 (51.9) |  |
| **Number of**  **prenatal appointments** | None | 51,355 (0.9) | 5,152 (0.2) | 0.299 | 3,989 (0.2) | 4,942 (0.3) | 0.268 |
|  | 0-3 appointments | 476,316 (8.0) | 82,428 (3.5) |  | 73,239 (3.7) | 78,168 (4.0) |  |
|  | 4-6 appointments | 1,656,989 (27.8) | 486,816 (20.5) |  | 430,592 (21.9) | 446,410 (22.7) |  |
|  | 7+ appointments | 3,774,213 (63.3) | 1,795,974 (75.8) |  | 1,462,02 (74.2) | 144,032 (73.1) |  |
| **Birth weight** | <1000 | 2,340 (0.0) | 938 (0.0) | 0.181 | 608 (0.0) | 763 (0.0) | 0.015 |
| **(grams)** | 1000-1199 | 736 (0.0) | 343 (0.0) |  | 208 (0.0) | 291 (0.0) |  |
|  | 1200-1399 | 945 (0.0) | 466 (0.0) |  | 305 (0.0) | 368 (0.0) |  |
|  | 1400-1599 | 1,549 (0.0) | 893 (0.0) |  | 562 (0.0) | 660 (0.0) |  |
|  | 1600-1799 | 3,622 (0.1) | 1,957 (0.1) |  | 1,316 (0.1) | 1,511 (0.1) |  |
|  | 1800-1999 | 9,564 (0.2) | 4,504 (0.2) |  | 3,118 (0.2) | 3,640 (0.2) |  |
|  | 2000-2199 | 31,841 (0.5) | 12,134 (0.5) |  | 9,565 (0.5) | 10,328 (0.5) |  |
|  | 2200-2399 | 88,042 (1.5) | 29,864 (1.2) |  | 25,427 (1.3) | 26,149 (1.3) |  |
|  | 2400-2599 | 221,751 (3.7) | 71,565 (3.0) |  | 62,628 (3.2) | 63,081 (3.2) |  |
|  | 2600-2799 | 486,188 (8.0) | 157,121 (6.6) |  | 136,530 (6.9) | 137,856 (7.0) |  |
|  | 2800-2999 | 836,471 (13.8) | 282,832 (11.8) |  | 244,311 (12.4) | 245,510 (12.5) |  |
|  | 3000-3199 | 1,126,784 (18.6) | 401,189 (16.7) |  | 343,119 (17.4) | 342,518 (17.4) |  |
|  | 3200-3399 | 1,138,569 (18.8) | 436,823 (18.2) |  | 369,051 (18.7) | 365,449 (18.6) |  |
|  | 3400-3599 | 914,046 (15.1) | 379,325 (15.8) |  | 313,217 (15.9) | 309,732 (15.7) |  |
|  | 3600-3799 | 600,353 (9.9) | 275,670 (11.5) |  | 219,299 (11.1) | 218,239 (11.1) |  |
|  | 3800-3999 | 331,482 (5.5) | 171,116 (7.1) |  | 128,458 (6.5) | 129,598 (6.6) |  |
|  | 4000-4199 | 152,368 (2.5) | 90,857 (3.8) |  | 63,100 (3.2) | 64,211 (3.3) |  |
|  | 4200-4399 | 63,422 (1.0) | 43,657 (1.8) |  | 28,450 (1.4) | 28,804 (1.5) |  |
|  | 4400-4599 | 24,778 (0.4) | 19,848 (0.8) |  | 12,160 (0.6) | 12,307 (0.6) |  |
|  | 4600-4799 | 9,035 (0.1) | 8,465 (0.4) |  | 4,906 (0.2) | 5,086 (0.3) |  |
|  | 4800-4999 | 3,548 (0.1) | 3,618 (0.2) |  | 2,060 (0.1) | 2,146 (0.1) |  |
|  | 5000+ | 2,292 (0.0) | 2,562 (0.1) |  | 1,445 (0.1) | 1,596 (0.1) |  |
| **Robson group** | Robson 1 | 1,553,898 (25.7) | 1,336,141 (55.8) | 0.689 | 962,169 (48.8) | 967,255 (49.1) | 0.006 |
|  | Robson 2a | 896,049 (14.8) | 310,326 (25.8) |  | 295,322 (15.0) | 295,790 (15.0) |  |
|  | Robson 3 | 2,576,452 (42.5) | 618,340 (12.9) |  | 586,750 (29.8) | 581,251 (29.5) |  |
|  | Robson 4a | 1,028,958 (17.0) | 131,800 (5.5) |  | 125,602 (6.4)) | 125,547 (6.4) |  |
|  | Robson 5 | - | - |  | - | - |  |
|  | Robson 6 | - | - |  | - | - |  |
|  | Robson 7 | - | - |  | - | - |  |
|  | Robson 8 | - | - |  | - | - |  |
|  | Robson 9 | - | - |  | - | - |  |
|  | Robson 10 | - | - |  | - | - |  |
| **Municipality HDI** | Very High | 1,246,989 (20.6) \| | 399754 (16.7) | 0.167 | 345,552 (17.5) | 347,207 (17.6) | 0.011 |
|  | High | 3,066,036 (50.6) | 1360553 (56.8) |  | 1,084,578 (55.1) | 1,074,544 (54.5) |  |
|  | Medium | 1,578,589 (26.1) | 608491 (25.4) |  | 514,566 (26.1) | 521,925 (26.5) |  |
|  | Low | 163,723 (2.7) | 27809 (1.2) |  | 25,147 (1.3) | 26,167 (1.3) |  |

CD: caesarean delivery; HDI: Human Development Index

| **Table C. Standardized mean differences (SMD) before and after matching for the matching covariates, Robson group 5.** | | | | | | | |
| --- | --- | --- | --- | --- | --- | --- | --- |
|  |  | **Before matching** | | | **After matching** | | |
|  |  | **Vaginal delivery** | **CD** |  | **Vaginal delivery** | **CD** |  |
|  |  | **N(%)** | **N(%)** | **SMD** | **N(%)** | **N(%)** | **SMD** |
| **Maternal age** | < 14 years | 45 (0.0) | 240 (0.0) | 0.330 | 26 (0.0) | 40 (0.0) | 0.034 |
|  | 14-15 years | 1,210 (0.2) | 4,471 (0.1) |  | 972 (0.2) | 1,106 (0.2) |  |
|  | 16-17 years | 10,774 (1.8) | 35,357 (1.0) |  | 10,059 (1.8) | 9,924 (1.8) |  |
|  | 18-19 years | 33,212 (5.7) | 112,069 (3.2) |  | 31,902 (5.8) | 30,818 (5.7) |  |
|  | 20-21 years | 55,603 (9.5) | 206,810 (5.9) |  | 53,608 (9.8) | 51,827 (9.5) |  |
|  | 22-23 years | 68,109 (11.7) | 282,113 (8.1) |  | 65,130 (11.9) | 63,736 (11.7) |  |
|  | 24-25 years | 70,883 (12.2) | 332,113 (9.5) |  | 67,475 (12.4) | 66,356 (12.2) |  |
|  | 26-27 years | 68,197 (11.7) | 368,943 (10.6) |  | 64,387 (11.8) | 63,850 (11.7) |  |
|  | 28-29 years | 63,162 (10.8) | 395,241 (11.3) |  | 58,984 (10.8) | 59,117 (10.8) |  |
|  | 30-31 years | 57,720 (9.9) | 409,893 (11.7) |  | 53,804 (9.9) | 54,112 (9.9) |  |
|  | 32-33 years | 49,283 (8.5) | 391,454 (11.2) |  | 45,381 (8.3) | 46,177 (8.5) |  |
|  | 34-35 years | 39,928 (6.8) | 351,208 (10.1) |  | 36,593 (6.7) | 37,590 (6.9) |  |
|  | 36-37 years | 29,608 (5.1) | 271,734 (7.8) |  | 26,771 (4.9) | 27,697 (5.1) |  |
|  | 38-39 years | 18,876 (3.2) | 177,624 (5.1) |  | 16,820 (3.1) | 17,728 (3.3) |  |
|  | 40-41 years | 10,304 (1.8) | 96,740 (2.8) |  | 8,875 (1.6) | 9,658 (1.8) |  |
|  | 42-43 years | 4,480 (0.8) | 39,486 (1.1) |  | 3,525 (0.6) | 4,140 (0.8) |  |
|  | 44-45 years | 1,302 (0.2) | 11,508 (0.3) |  | 914 (0.2) | 1,195 (0.2) |  |
|  | 46-47 years | 285 (0.0) | 2,408 (0.1) |  | 163 (0.0) | 257 (0.0) |  |
|  | 48-49 years | 66 (0.0) | 457 (0.0) |  | 23 (0.0) | 62 (0.0) |  |
|  | 50+ years | 54 (0.0) | 318 (0.0) |  | 24 (0.0) | 46 (0.0) |  |
| **Marital status** | Single | 264,352 (45.8) | 1,108,773 (32.0) | 0.284 | 250,733 (46.0) | 247,430 (45.4) | 0.025 |
|  | Widow | 303,813 (52.6) | 2,275,825 (65.8) |  | 287,019 (52.6) | 288,919 (53.0) |  |
|  | Divorced | 1,295 (0.2) | 8,527 (0.2) |  | 814 (0.1) | 1,225 (0.2) |  |
|  | Married/union | 8,258 (1.4) | 66,926 (1.9) |  | 6,870 (1.3) | 7,862 (1.4) |  |
| **Maternal education** | none | 4,241 (0.7) | 8,246 (0.2) | 0.487 | 2,982 (0.5) | 3,710 (0.7) | 0.022 |
|  | 1-3 years | 25,122 (4.4) | 7,1247 (2.1) |  | 21,708 (4.0) | 23,138 (4.2) |  |
|  | 4-7 years | 153,981 (26.7) | 547,339 (15.9) |  | 145,539 (26.7) | 144,406 (26.5) |  |
|  | 8-12 years | 342,120 (59.4) | 1,979,900 (57.4) |  | 327,640 (60.1) | 325,916 (59.8) |  |
|  | 12+ years | 50,851 (8.8) | 843,321 (24.4) |  | 47,567 (8.7) | 48,266 (8.8) |  |
| **Maternal ethnicity** | White | 184,482 (32.6) | 1,532,594 (45.4) | 0.278 | 178,789 (32.8) | 180,001 (33.0) | 0.025 |
|  | Black | 38,371 (6.8) | 1,66,425 (4.9) |  | 35,596 (6.5) | 36,633 (6.7) |  |
|  | Asian | 2,045 (0.4) | 13,933 (0.4) |  | 1,550 (0.3) | 1,962 (0.4) |  |
|  | Mixed race | 336,532 (59.4) | 1,654,824 (49.0) |  | 325,683 (59.7) | 322,155 (59.1) |  |
|  | Indigenous | 5,283 (0.9) | 8,438 (0.2) |  | 3,818 (0.7) | 4,685 (0.9) |  |
| **Year of birth** | 2012 | 66,047 (11.3) | 414,718 (11.9) | 0.057 | 60,743 (11.1) | 61,332 (11.2) | 0.008 |
|  | 2013 | 69,905 (12.0) | 456,735 (13.1) |  | 63,895 (11.7) | 64,780 (11.9) |  |
|  | 2014 | 76,177 (13.1) | 492,299 (14.1) |  | 70,447 (12.9) | 71,082 (13.0) |  |
|  | 2015 | 87,637 (15.0) | 512,932 (14.7) |  | 81,988 (15.0) | 81,856 (15.0) |  |
|  | 2016 | 89,068 (15.3) | 504,317 (14.4) |  | 83,864 (15.4) | 83,239 (15.3) |  |
|  | 2017 | 94,879 (16.3) | 538,701 (15.4) |  | 89,494 (16.4) | 89,005 (16.3) |  |
|  | 2018 | 99,388 (17.0) | 570,485 (16.3) |  | 95,005 (17.4) | 94,142 (17.3) |  |
| **Sex of the newborn** | Female | 293,561 (50.3) | 1,699,421 (48.7) | 0.033 | 275,031 (50.4) | 274,691 (50.4) | 0.001 |
|  | Male | 289,490 (49.7) | 1,790,406 (51.3) |  | 270,405 (49.6) | 270,745 (49.6) |  |
| **Number of**  **prenatal appointments** | None | 7,700 (1.4) | 10,055 (0.3) | 0.344 | 6,042 (1.1) | 7,199 (1.3) | 0.020 |
|  | 0-3 appointments | 56,641 (9.9) | 144,921 (4.2) |  | 53,039 (9.7) | 53,950 (9.9) |  |
|  | 4-6 appointments | 162,869 (28.6) | 718,398 (20.8) |  | 155,779 (28.6) | 155,275 (28.5) |  |
|  | 7+ appointments | 342,882 (60.1) | 2,573,226 (74.7) |  | 330,576 (60.6) | 329,012 (60.3) |  |
| **Birth weight** | <1000 | 283 (0.0) | 1249 (0.0) | 0.180 | 157 (0.0) | 235 (0.0) | 0.031 |
| **(grams)** | 1000-1199 | 89 (0.0) | 550 (0.0) |  | 45 (0.0) | 74 (0.0) |  |
|  | 1200-1399 | 103 (0.0) | 520 (0.0) |  | 43 (0.0) | 89 (0.0) |  |
|  | 1400-1599 | 186 (0.0) | 1,005 (0.0) |  | 95 (0.0) | 163 (0.0) |  |
|  | 1600-1799 | 395 (0.1) | 1,884 (0.1) |  | 218 (0.0) | 353 (0.1) |  |
|  | 1800-1999 | 1,068 (0.2) | 4,476 (0.1) |  | 675 (0.1) | 950 (0.2) |  |
|  | 2000-2199 | 3,209 (0.6) | 12,238 (0.4) |  | 2,390 (0.4) | 2,874 (0.5) |  |
|  | 2200-2399 | 8,329 (1.4) | 32,145 (0.9) |  | 7,006 (1.3) | 7,576 (1.4) |  |
|  | 2400-2599 | 19,757 (3.4) | 83,012 (2.4) |  | 17,651 (3.2) | 18,106 (3.3) |  |
|  | 2600-2799 | 42,840 (7.4) | 199,541 (5.7) |  | 39,539 (7.2) | 39,738 (7.3) |  |
|  | 2800-2999 | 74,967 (12.9) | 389,450 (11.2) |  | 70,050 (12.8) | 69,954 (12.8) |  |
|  | 3000-3199 | 104,116 (17.9) | 586,296 (16.8) |  | 98,095 (18.0) | 97,687 (17.9) |  |
|  | 3200-3399 | 110,208 (18.9) | 657,664 (18.8) |  | 104,386 (19.1) | 103,433 (19.0) |  |
|  | 3400-3599 | 91,742 (15.7) | 578,306 (16.6) |  | 87,206 (16.0) | 86,275 (15.8) |  |
|  | 3600-3799 | 62,232 (10.7) | 421,340 (12.1) |  | 59,102 (10.8) | 58,540 (10.7) |  |
|  | 3800-3999 | 3,5270 (6.1) | 258,536 (7.4) |  | 33,392 (6.1) | 33,206 (6.1) |  |
|  | 4000-4199 | 16,586 (2.8) | 137,818 (4.0) |  | 15,499 (2.8) | 15,570 (2.9) |  |
|  | 4200-4399 | 6,976 (1.2) | 67,045 (1.9) |  | 6,306 (1.2) | 6,533 (1.2) |  |
|  | 4400-4599 | 2,762 (0.5) | 31,052 (0.9) |  | 2,405 (0.4) | 2,581 (0.5) |  |
|  | 4600-4799 | 963 (0.2) | 13,681 (0.4) |  | 732 (0.1) | 898 (0.2) |  |
|  | 4800-4999 | 390 (0.1) | 6,228 (0.2) |  | 270 (0.0) | 362 (0.1) |  |
|  | 5000+ | 252 (0.0) | 4,972 (0.1) |  | 174 (0.0) | 239 (0.0) |  |
| **Robson group** | Robson 1 | - | - |  | - | - | - |
|  | Robson 2a | - | - |  | - | - |  |
|  | Robson 3 | - | - |  | - | - |  |
|  | Robson 4a | - | - |  | - | - |  |
|  | Robson 5 | - | - |  | - | - |  |
|  | Robson 6 | - | - |  | - | - |  |
|  | Robson 7 | - | - |  | - | - |  |
|  | Robson 8 | - | - |  | - | - |  |
|  | Robson 9 | - | - |  | - | - |  |
|  | Robson 10 | - | - |  | - | - |  |
| **Municipality HDI** | Very High | 136,928 (23.5) | 75,6831 (21.7) | 0.159 | 130,281 (23.9) | 129,816 (23.8) | 0.017 |
|  | High | 316,653 (54.3) | 2,107,070 (60.4) |  | 298,686 (54.8) | 296,456 (54.4) |  |
|  | Medium | 118,782 (20.4) | 604,848 (17.3) |  | 107,896 (19.8) | 109,590 (20.1) |  |
|  | Low | 10,735 (1.8) | 21,438 (0.6) |  | 8,573 (1.6) | 9,574 (1.8) |  |

CD: caesarean delivery; HDI: Human Development Index

| **Table D. Standardized mean differences (SMD) before and after matching for the matching covariates, Robson groups 6-10.** | | | | | | | |
| --- | --- | --- | --- | --- | --- | --- | --- |
|  |  | **Before matching** | | | **After matching** | | |
|  |  | **Vaginal delivery** | **CD** |  | **Vaginal delivery** | **CD** |  |
|  |  | **N(%)** | **N(%)** | **SMD** | **N(%)** | **N(%)** | **SMD** |
| **Maternal age** | < 14 years | 5,297 (0.5) | 3,167 (0.2) | 01.509 | 2,141 (0.3) | 2,174 (0.3) | 0.049 |
|  | 14-15 years | 5,0420 (4.7) | 31,200 (1.7) |  | 20,113 (2.8) | 17,298 (2.4) |  |
|  | 16-17 years | 112,484(10.5) | 83,064 (4.4) |  | 51,006 (7.2) | 44,892 (6.3) |  |
|  | 18-19 years | 13,4045(12.5) | 123,718 (6.6) |  | 70,693 (9.9) | 68,265 (9.6) |  |
|  | 20-21 years | 126,732(11.8) | 146,227 (7.8) |  | 76,332(10.7) | 77,109(10.8) |  |
|  | 22-23 years | 108,937(10.2) | 153,971 (8.2) |  | 73,161(10.3) | 74,135(10.4) |  |
|  | 24-25 years | 96,393 (9.0) | 163,662 (8.7) |  | 69,986 (9.8) | 70,892 (9.9) |  |
|  | 26-27 years | 87,365 (8.1) | 175,221 (9.3) |  | 66,583 (9.3) | 67,744 (9.5) |  |
|  | 28-29 years | 79,398 (7.4) | 185,707 (9.9) |  | 62,591 (8.8) | 63,901 (9.0) |  |
|  | 30-31 years | 71,885 (6.7) | 191,228(10.2) |  | 58,350 (8.2) | 59,145 (8.3) |  |
|  | 32-33 years | 60,874 (5.7) | 178,474 (9.5) |  | 50,066 (7.0) | 50,727 (7.1) |  |
|  | 34-35 years | 50,060 (4.7) | 157,994 (8.4) |  | 41,119 (5.8) | 42,269 (5.9) |  |
|  | 36-37 years | 38,071 (3.6) | 121,947 (6.5) |  | 31,270 (4.4) | 31,997 (4.5) |  |
|  | 38-39 years | 25,217 (2.4) | 82,314 (4.4) |  | 20,314 (2.8) | 21,382 (3.0) |  |
|  | 40-41 years | 14,903 (1.4) | 47,523 (2.5) |  | 11,737 (1.6) | 12,537 (1.8) |  |
|  | 42-43 years | 6,926 (0.6) | 21,434 (1.1) |  | 5,303 (0.7) | 5,813 (0.8) |  |
|  | 44-45 years | 2,350 (0.2) | 7,627 (0.4) |  | 1,596 (0.2) | 1,927 (0.3) |  |
|  | 46-47 years | 646 (0.1) | 2,278 (0.1) |  | 386 (0.1) | 506 (0.1) |  |
|  | 48-49 years | 136 (0.0) | 703 (0.0) |  | 83 (0.0) | 98 (0.0) |  |
|  | 50+ years | 146 (0.0) | 549 (0.0) |  | 70 (0.0) | 89 (0.0) |  |
| **Marital status** | Single | 528,806(50.0) | 701,423(37.7) | 0.253 | 3,27,680(46.0) | 324,019(45.5) | 0.016 |
|  | Widow | 519,586(49.1) | 1,128,975(60.6) |  | 377,367(52.9) | 380,037(53.3) |  |
|  | Divorced | 1,867 (0.2) | 3,867 (0.2) |  | 1,192 (0.2) | 1,430 (0.2) |  |
|  | Married/union | 8,388 (0.8) | 27,282 (1.5) |  | 6,661 (0.9) | 7,414 (1.0) |  |
| **Maternal education** | none | 12,368 (1.2) | 7,426 (0.4) | 0.522 | 4,377 (0.6) | 4,216 (0.6) | 0.028 |
|  | 1-3 years | 52,151 (4.9) | 45,031 (2.4) |  | 24,733 (3.5) | 24,566 (3.4) |  |
|  | 4-7 years | 289,430(27.5) | 293,966(15.9) |  | 158,090(22.2) | 150,081(21.1) |  |
|  | 8-12 years | 611,922(58.1) | 1,040,675(56.2) |  | 444,708(62.4) | 452,496(63.5) |  |
|  | 12+ years | 88,051 (8.4) | 465,617(25.1) |  | 80,992(11.4) | 81,541(11.4) |  |
| **Maternal ethnicity** | White | 282,542(27.4) | 794,294(43.9) | 0.372 | 238,930(33.5) | 246,873(34.6) | 0.043 |
|  | Black | 66,387 (6.4) | 97,550 (5.4) |  | 43,162 (6.1) | 46,446 (6.5) |  |
|  | Asian | 3,947 (0.4) | 7,836 (0.4) |  | 2,608 (0.4) | 3,020 (0.4) |  |
|  | Mixed race | 659,747(63.9) | 903,124(49.9) |  | 423,740(59.4) | 413,519(58.0) |  |
|  | Indigenous | 20,076 (1.9) | 6,806 (0.4) |  | 4,460 (0.6) | 3,042 (0.4) |  |
| **Year of birth** | 2012 | 163,142(15.2) | 263,865(14.1) | 0.045 | 102,891(14.4) | 103,525(14.5) | 0.007 |
|  | 2013 | 156,755(14.6) | 266,147(14.2) |  | 101,570(14.2) | 102,266(14.3) |  |
|  | 2014 | 154,944(14.4) | 271,768(14.5) |  | 102,282(14.3) | 103,144(14.5) |  |
|  | 2015 | 154,844(14.4) | 268,292(14.3) |  | 102,400(14.4) | 101,401(14.2) |  |
|  | 2016 | 149,978(14.0) | 264,769(14.1) |  | 100,779(14.1) | 99,897(14.0) |  |
|  | 2017 | 146,527(13.7) | 268,705(14.3) |  | 100,410(14.1) | 100,245(14.1) |  |
|  | 2018 | 146136(13.6) | 274462(14.6) |  | 102,568(14.4) | 102,422(14.4) |  |
| **Sex of the newborn** | Female | 507,775(47.4) | 915,897(48.8) | 0.028 | 335,005(47.0) | 336,943(47.3) | 0.005 |
|  | Male | 563,955(52.6) | 961,094(51.2) |  | 377,895(53.0) | 375,957(52.7) |  |
| **Number of**  **prenatal appointments** | None | 19,260 (1.9) | 8,673 (0.5) | 0.586 | 6,541 (0.9) | 5,445 (0.8) | 0.068 |
|  | 0-3 appointments | 195,478(18.8) | 126,814 (6.9) |  | 85,876(12.0) | 72,016(10.1) |  |
|  | 4-6 appointments | 429,910(41.5) | 518,862(28.2) |  | 28,1504(39.5) | 281,465(39.5) |  |
|  | 7+ appointments | 392,519(37.8) | 118,4940(64.4) |  | 338,979(47.5) | 353,974(49.7) |  |
| **Birth weight** | <1000 | 52,826 (4.9) | 45,058 (2.4) | 0.190 | 28,617 (4.0) | 24,712 (3.5) | 0.032 |
| **(grams)** | 1000-1199 | 16,319 (1.5) | 29,247 (1.6) |  | 11,635 (1.6) | 11,914 (1.7) |  |
|  | 1200-1399 | 18,592 (1.7) | 37,442 (2.0) |  | 13,718 (1.9) | 14,120 (2.0) |  |
|  | 1400-1599 | 23,200 (2.2) | 48,021 (2.6) |  | 17,520 (2.5) | 17,896 (2.5) |  |
|  | 1600-1799 | 31,752 (3.0) | 63,341 (3.4) |  | 23,952 (3.4) | 24,107 (3.4) |  |
|  | 1800-1999 | 45,040 (4.2) | 85,134 (4.5) |  | 33,165 (4.7) | 33,558 (4.7) |  |
|  | 2000-2199 | 66,486 (6.2) | 116,413 (6.2) |  | 47,387 (6.6) | 47,251 (6.6) |  |
|  | 2200-2399 | 92,601 (8.7) | 152,929 (8.1) |  | 63,298 (8.9) | 63,543 (8.9) |  |
|  | 2400-2599 | 117,248(11.0) | 184,790 (9.8) |  | 765,75(10.7) | 77,175(10.8) |  |
|  | 2600-2799 | 129,487(12.1) | 206,122(11.0) |  | 82,922(11.6) | 83,746(11.7) |  |
|  | 2800-2999 | 129,012(12.1) | 214,180(11.4) |  | 82,908(11.6) | 82,148(11.5) |  |
|  | 3000-3199 | 115,215(10.8) | 203,789(10.9) |  | 73,271(10.3) | 73,102(10.3) |  |
|  | 3200-3399 | 91,558 (8.6) | 175,170 (9.3) |  | 60,268 (8.5) | 60,358 (8.5) |  |
|  | 3400-3599 | 64,620 (6.0) | 130,620 (7.0) |  | 43,553 (6.1) | 43,724 (6.1) |  |
|  | 3600-3799 | 38,763 (3.6) | 86,044 (4.6) |  | 27,531 (3.9) | 27,701 (3.9) |  |
|  | 3800-3999 | 20,722 (1.9) | 50,213 (2.7) |  | 15,169 (2.1) | 15,424 (2.2) |  |
|  | 4000-4199 | 9,164 (0.9) | 25,362 (1.4) |  | 6,821 (1.0) | 7,215 (1.0) |  |
|  | 4200-4399 | 3,826 (0.4) | 12,457 (0.7) |  | 2,910 (0.4) | 3,184 (0.4) |  |
|  | 4400-4599 | 1,479 (0.1) | 5,757 (0.3) |  | 1,073 (0.2) | 1,248 (0.2) |  |
|  | 4600-4799 | 506 (0.0) | 2,426 (0.1) |  | 336 (0.0) | 428 (0.1) |  |
|  | 4800-4999 | 201 (0.0) | 1,143 (0.1) |  | 126 (0.0) | 168 (0.0) |  |
|  | 5000+ | 215 (0.0) | 1,052 (0.1) |  | 145 (0.0) | 178 (0.0) |  |
| **Robson group** | Robson 1 | - | - | 0.850 | - | - | 0.031 |
|  | Robson 2a | - | - |  | - | - |  |
|  | Robson 3 | - | - |  | - | - |  |
|  | Robson 4a | - | - |  | - | - |  |
|  | Robson 5 | - | - |  | - | - |  |
|  | Robson 6 | 24,911 (2.3) | 234,081(12.5) |  | 2,1731 (3.0) | 22,703 (3.2) |  |
|  | Robson 7 | 53,920 (5.0) | 317,616(16.9) |  | 46,314 (6.5) | 47,545 (6.7) |  |
|  | Robson 8 | 71,552 (6.7) | 352,631(18.8) |  | 57,448 (8.1) | 62,930 (8.8) |  |
|  | Robson 9 | 1,318 (0.1) | 40,564 (2.2) |  | 1,307 (0.2) | 1,172 (0.2) |  |
|  | Robson 10 | 920,625(85.9) | 933,116(49.7) |  | 586,100(82.2) | 578,550(81.2) |  |
| **Municipality HDI** | Very High | 186,531(17.4) | 438,730(23.4) | 0.370 | 141,566(19.9) | 148,720(20.9) | 0.057 |
|  | High | 559,376(52.2) | 1,143,298(60.9) |  | 418,348(58.7) | 425,323(59.7) |  |
|  | Medium | 292,682(27.3) | 282,480(15.0) |  | 144,357(20.2) | 132,755(18.6) |  |
|  | Low | 33,730 (3.1) | 13,500 (0.7) |  | 8,629 (1.2) | 6,102 (0.9) |  |

CD: caesarean delivery; HDI: Human Development Index

| **Table E. Logistic regression estimates of the odds of delivery by CD in Robson groups 1-4 or by vaginal delivery in Robson group 5 and groups 6-10** | | | | | | | | | | |
| --- | --- | --- | --- | --- | --- | --- | --- | --- | --- | --- |
|  |  | **Robson groups 1-4** | | **Robson group 5** | | | **Robson groups 6-10** | | | |
|  |  | **Odds ratios** | **p-value^1^** | **Odds ratios** | **p-value^1^** | | **Odds ratios** | | **p-value^1^** | |
|  | **Intercept** | 0.93 (0.92-0.94) | <0.001 | 0.14 (0.13- 0.14) | <0.001 | | 0.08 (0.08- 0.08) | | <0.001 | |
| **Maternal age** | < 14 years | 1.11 (1.07-1.14) | <0.001 | 0.51 (0.36-0.72) | <0.001 | | 1.05 (1.00-1.11) | | 0.050 | |
|  | 14-15 years | 0.81 (0.80-0.82) | <0.001 | 0.75 (0.70-0.80) | <0.001 | | 1.31 (1.29-1.34 | | <0.001 | |
|  | 16-17 years | 0.79 (0.78-0.79) | <0.001 | 0.93 (0.91-0.95) | <0.001 | | 1.27 (1.25-1.29) | | <0.001 | |
|  | 18-19 years | 0.87 (0.86-0.88) | <0.001 | 1.01 (0.99-1.02) | 0.439 | | 1.14 (1.13-1.15) | | <0.001 | |
|  | 20-21 years | 1 |  | 1 |  | | 1 | |  | |
|  | 22-23 years | 1.13 (1.12-1.14) | <0.001 | 0.98 (0.96-0.99) | 0.001 | | 0.89 (0.88-0.90) | | <0.001 | |
|  | 24-25 years | 1.29 (1.28-1.30) | <0.001 | 0.94 (0.92-0.95) | <0.001 | | 0.80 (0.79-0.81) | | <0.001 | |
|  | 26-27 years | 1.43 (1.42- 1.45) | <0.001 | 0.88 (0.86-0.89) | <0.001 | | 0.73 (0.72-0.74) | | <0.001 | |
|  | 28-29 years | 1.58 (1.57-1.60 | <0.001 | 0.82 (0.80-0.82) | <0.001 | | 0.68 (0.67-0.69) | | <0.001 | |
|  | 30-31 years | 1.73 (1.72- 1.74) | <0.001 | 0.77 (0.76-0.78) | <0.001 | | 0.63 (0.62-0.64) | | <0.001 | |
|  | 32-33 years | 1.84 (1.83-1.86) | <0.001 | 0.72 (0.71-0.73) | <0.001 | | 0.59 (0.58-0.60) | | <0.001 | |
|  | 34-35 years | 2.00 (1.98-2.01) | <0.001 | 0.68 (0.67-0.68) | <0.001 | | 0.55 (0.54-0.56) | | <0.001 | |
|  | 36-37 years | 2.15 (2.13-2.17) | <0.001 | 0.64 (0.63-0.65) | <0.001 | | 0.53 (0.52-0.53) | | <0.001 | |
|  | 38-39 years | 2.34 (2.31-2.37) | <0.001 | 0.61 (0.60-0.62) | <0.001 | | 0.49 (0.48-0.50) | | <0.001 | |
|  | 40-41 years | 2.57 (2.53- 2.62) | <0.001 | 0.58 (0.57-0.60) | <0.001 | | 0.46 (0.45-0.47) | | <0.001 | |
|  | 42-43 years | 2.80 (2.74- 2.87) | <0.001 | 0.58 (0.56-0.60) | <0.001 | | 0.43 (0.42-0.45) | | <0.001 | |
|  | 44-45 years | 2.98 (2.86-3.10) | <0.001 | 0.54 (0.51-0.58) | <0.001 | | 0.40 (0.38-0.43) | | <0.001 | |
|  | 46-47 years | 3.13 (2.88-3.40) | <0.001 | 0.51 (0.45-0.59) | <0.001 | | 0.40 (0.36-0.45) | | <0.001 | |
|  | 48-49 years | 2.81 (2.38-3.32) | <0.001 | 0.69 (0.53-0.91) | 0.009 | | 0.32 (0.25-0.40) | | <0.001 | |
|  | 50+ years | 1.77 (1.47-2.14) | <0.001 | 0.78 (0.56-1.07) | 0.128 | | 0.38 (0.29-0.48) | | <0.001 | |
| **Marital status** | Single | 0.88 (0.87-088) | <0.001 | 1.30 (1.30-1.31) | <0.001 | | 1.13 (1.12-1.14) | | <0.001 | |
|  | Widow | 0.99 (0.95-1.03) | 0.688 | 1.06 (1.00-1.13) | 0.050 | | 1.03 (0.97-1.10) | | 0.299 | |
|  | Divorced | 1.02 (1.00-1.03) | 0.086 | 1.02 (1.00-1.05) | 0.076 | | 0.89 (0.87-0.91) | | <0.001 | |
|  | Married/union | 1 |  | 1 |  | | 1 | |  | |
| **Maternal education** | None | 0.61 (0.60-0.63) | <0.001 | 2.56 (2.46-2.67) | <0.001 | | 2.18 (2.10-2.26) | | <0.001 | |
|  | 1 - 3 years | 0.69 (0.69-0.70) | <0.001 | 1.94 (1.91-1.97) | <0.001 | | 1.74 (1.72-1.77) | | <0.001 | |
|  | 4 - 7 years | 0.82 (0.82-0.83) | <0.001 | 1.46 (1.45-1.47) | <0.001 | | 1.32 (1.31-1.33) | | <0.001 | |
|  | 8 - 12 years | 1 |  | 1 |  | | 1 | |  | |
|  | 12 + years | 1.57 (1.57-1.58) | <0.001 | 0.44 (0.44-0.45) | <0.001 | | 0.53 (0.53-0.54) | | <0.001 | |
| **Maternal ethnicity** | White | 1 |  | 1 |  | | 1 | |  | |
|  | Black | 0.76 (0.75-0.77) | <0.001 | 1.36 (1.34-1.38) | <0.001 | | 1.30 (1.29-1.32) | | <0.001 | |
|  | Asian | 0.74 (0.72-0.76) | <0.001 | 1.16 (1.11-1.22) | <0.001 | | 1.38 (1.32-1.44) | | <0.001 | |
|  | Mixed race | 0.87 (0.87-0.88) | <0.001 | 1.280 (1.27-1.28) | <0.001 | | 1.29 (1.28-1.30) | | <0.001 | |
|  | Indigenous | 0.39 (0.38-0.40) | <0.001 | 2.98 (2.87-3.10) | <0.001 | | 2.71 (2.62-2.80) | | <0.001 | |
| **Year of birth** | 2012 | 1 |  | 1 |  | | 1 | |  | |
|  | 2013 | 1.01 (1.00-1.01) | 0.055 | 0.98 (0.97-0.99) | <0.001 | | 0.95 (0.94-0.96) | | <0.001 | |
|  | 2014 | 0.96 (0.95-0.96) | <0.001 | 1.02 (1.01-1.03) | <0.001 | | 0.98 (0.96-0.98) | | <0.001 | |
|  | 2015 | 0.84 (0.83-0.84) | <0.001 | 1.16 (1.15-1.17) | <0.001 | | 1.04 (1.03-1.05) | | <0.001 | |
|  | 2016 | 0.81 (0.81-0.82) | <0.001 | 1.21 (1.20-1.23) | <0.001 | | 1.03 (1.02-1.05) | | <0.001 | |
|  | 2017 | 0.79 (0.78-0.79) | <0.001 | 1.24 (1.23-1.26) | <0.001 | | 1.02 (1.01-1.03) | | <0.001 | |
|  | 2018 | 0.77 (0.77-0.78) | <0.001 | 1.26 (01.25-1.27) | <0.001 | | 1.03 (1.02-1.05) | | <0.001 | |
| **Sex of the newborn** | Female | 1 |  | 1 |  | | 1 | |  | |
|  | Male | 1.06 (1.05-1.06) | <0.001 | 0.97 (0.96-0.98) | <0.001 | | 1.01 (1.01-1.02) | | <0.001 | |
| **Number of**  **prenatal appointments** | None | 0.34 (0.33-0.35) | <0.001 | 3.76 (3.65-3.88) | <0.001 | | 4.17 (4.05-4.30) | | <0.001 | |
|  | 0-3 appointments | 0.55 (0.55-0.56) | <0.001 | 2.04 (2.02-2.07) | <0.001 | | 2.56 (2.53-2.58) | | <0.001 | |
|  | 4-6 appointments | 0.76 (0.75-0.76) | <0.001 | 1.38 (1.37-1.39) | <0.001 | | 1.61 (1.60-1.62) | | <0.001 | |
|  | 7+ appointments | 1 |  | 1 |  | | 1 | |  | |
| **Birth weight** | <1000 | 1.42 (1.31-1.55) | <0.001 | 0.92 (0.80-1.07) | 0.284 | | 1.64 (1.62-1.67) | | <0.001 | |
|  | 1000-1199 | 1.76 (1.52-2.03) | <0.001 | 0.66 (0.51-0.84) | 0.001 | | 0.68 (0.66-0.69) | | <0.001 | |
|  | 1200-1399 | 1.76 (1.55-1.99) | <0.001 | 0.77 (0.61-0.97) | 0.028 | | 0.60 (0.59-0.61) | | <0.001 | |
|  | 1400-1599 | 2.01 (1.82-2.21) | <0.001 | 0.71 (0.60-0.85) | <0.001 | | 0.61 (0.60-0.62 | | <0.001 | |
|  | 1600-1799 | 1.88 (1.77-2.00) | <0.001 | 0.83 (0.74-0.94) | 0.003 | | 0.67 (0.66-0.69) | | <0.001 | |
|  | 1800-1999 | 1.60 (1.53-1.66) | <0.001 | 0.96 (0.89-1.03) | 0.283 | | 0.76 (0.75-0.77) | | <0.001 | |
|  | 2000-2199 | 1.28 (1.25-1.31) | <0.001 | 1.06 (1.01-1.11) | 0.008 | | 0.86 (0.85-0.87) | | <0.001 | |
|  | 2200-2399 | 1.10 (1.08-1.11) | <0.001 | 1.09 (1.06-1.12) | <0.001 | | 0.94 (0.93-0.96) | | <0.001 | |
|  | 2400-2599 | 1.02 (1.00-1.03) | 0.003 | 1.05 (1.03-1.07) | <0.001 | | 1.00 (0.99-1.01) | | 0.818 | |
|  | 2600-2799 | 1 |  | 1 |  | | 1 | | <0.001 | |
|  | 2800-2999 | 1.05 (1.04-1.06) | <0.001 | 0.93 (0.92-0.94) | <0.001 | | 0.96 (0.95-0.97) | | <0.001 | |
|  | 3000-3199 | 1.13 (1.13-1.14) | <0.001 | 0.88 (0.86-0.88) | <0.001 | | 0.90 (0.89-0.91) | | <0.001 | |
|  | 3200-3399 | 1.26 (1.25-1.27) | <0.001 | 0.83 (0.82-0.84) | <0.001 | | 0.84 (0.83-0.85) | | <0.001 | |
|  | 3400-3599 | 1.42 (1.41-1.43) | <0.001 | 0.79 (0.79-0.80) | <0.001 | | 0.79 (0.78-0.80) | | <0.001 | |
|  | 3600-3799 | 1.64 (1.63-1.66) | <0.001 | 0.72 (0.71-0.74) | <0.001 | | 0.72 (0.70-0.73) | | <0.001 | |
|  | 3800-3999 | 1.95 (1.93-1.97) | <0.001 | 0.66 (0.65-0.67) | <0.001 | | 0.65 (0.64-0.66) | | <0.001 | |
|  | 4000-4199 | 2.38 (2.36-2.41) | <0.001 | 0.57 (0.56-0.59) | <0.001 | | 0.57 (0.55-0.58) | | <0.001 | |
|  | 4200-4399 | 2.86 (2.82-2.90) | <0.001 | 0.49 (0.47-0.50) | <0.001 | | 0.48 (0.46-0.50) | | <0.001 | |
|  | 4400-4599 | 3.51 (3.43-3.58) | <0.001 | 0.41 (0.39-0.43) | <0.001 | | 0.38 (0.36-0.41) | | <0.001 | |
|  | 4600-4799 | 4.23 (4.09-4.38) | <0.001 | 0.32 (0.30-0.34) | <0.001 | | 0.33 (0.30-0.37) | | <0.001 | |
|  | 4800-4999 | 4.73 (4.49-4.99) | <0.001 | 0.28 (0.25-0.31) | <0.001 | | 0.26 (0.21-0.30) | | <0.001 | |
|  | 5000+ | 5.40 (5.07-5.75) | <0.001 | 0.22 (0.20-0.26) | <0.001 | | 0.30 (0.25-0.36) | | <0.001 | |
| **Robson group** | Robson 1 | 1 |  | - |  | | - | |  | |
|  | Robson 2a | 0.22 (0.22-0.22) | <0.001 | - |  | | - | |  | |
|  | Robson 3 | 0.39 (0.38-0.39) | <0.001 | - |  | | - | |  | |
|  | Robson 4a | 0.10 (0.10-0.11) | <0.001 | - |  | | - | |  | |
|  | Robson 5 | - |  | - |  | | - | |  | |
|  | Robson 6 | - |  | - |  | | 1 | |  | |
|  | Robson 7 | - |  | - |  | | 1.71 (1.68-1.74) | | <0.001 | |
|  | Robson 8 | - |  | - |  | | 2.13 (2.10-2.17) | | <0.001 | |
|  | Robson 9 | - |  | - |  | | 0.26 (0.25-0.28) | | <0.001 | |
|  | Robson 10 | - |  | - |  | | 8.872 (8.74-9.00) | | <0.001 | |
| **Municipality HDI** | Very High | 0.60 (0.60-0.61) | <0.001 | 1.58 (01.57-1.59) | <0.001 | | 1.21 (1.20-1.22) | | <0.001 | |
|  | High (reference) | 1 |  | 1 | |  | | 1 | |  |
|  | Medium | 0.94 (0.94-0.95) | <0.001 | 1.04 (1.03-1.05) | <0.001 | | 1·71 (1·70-1·72) | | <0.001 | |
|  | Low | 0.55 (0.55-0.56) | <0.001 | 2.06 (2.01-2.11) | <0.001 | | 2.92 (2.85-2.99) | | <0.001 | |

HDI: Human Development Index CD- caesarean delivery

| **Table F. Mortality conditional on survival up to 6 days, 27 days and under 1 year, by mode of delivery in Robson groups 1-4 before and after propensity score matching (PSM), Brazil 2012-2018** | | | | | | | | | | | | | | |
| --- | --- | --- | --- | --- | --- | --- | --- | --- | --- | --- | --- | --- | --- | --- |
| **Robson Groups** |  | | **Survival from 7 days to 5 years** | |  |  | **Survival from 28 days to 5 years** | |  |  | **Survival from 1 to 5 years** | |  | |
|  | **Before PSM** | | | **After PSM** | | **Before PSM** | | **After PSM** | | **Before PSM** | | **After PSM** | | |
| **Robson Groups** | **^$^HR (95% CI)** | **p-value** | | **^$^HR (95% CI)** | **p-value** | **^$^HR (95% CI)** | **p-value** | **^$^HR (95% CI)** | **p-value** | **^$^HR (95% CI)** | **p-value** | **^$^HR (95% CI)** | **p-value** | |
| **1** | 0.89 (0.85-0.93) | <0.001 | | 1.18 (1.13-1.25) | <0.001 | 0.85 (0.81-0.89) | <0.001 | 1.14 (1.09-1.22) | <0.001 | 0.88 (0.81-0.95) | 0.001 | 1.18 (1.07-1.29) | <0.001 | |
| **2a** | 1.00 (0.93-1.08) | 0.999 | | 1.27 (1.15-1.40) | <0.001 | 0.94 (0.86-1.02) | 0.140 | 1.17 (1.05-1.30) | 0.006 | 0.86 (0.75-0.98) | 0.025 | 1.07 (0.90-1.27) | 0.445 | |
| **3** | 0.97 (0.93-1.01) | 0.160 | | 1.21 (1.14-1.28) | <0.001 | 0.92 (0.88-0.96) | <0.001 | 1.16 (1.09-1.24) | <0.001 | 0.87 (0.80-0.95) | 0.001 | 1.09 (0.98-1.21) | 0.122 | |
| **4a** | 1.15 (1.05-1.26) | 0.002 | | 1.32 (1.17-1.50) | <0.001 | 1.07 (0.97-1.19) | 0.172 | 1.21 (1.05-1.39) | 0.009 | 1.00 (0.85-1.19) | 0.985 | 1.04 (0.83-1.31) | 0.742 | |
| **1-4(2a,4a)** | 0.90 (0.88-0.92) | <0.001 | | 1.19 (1.15-1.23) | <0.001 | 0.85 (0.82-0.87) | <0.001 | 1.14 (1.10-1.19) | <0.001 | 0.85 (0.81-0.89) | <0.001 | 1.10 (1.04-1.17) | 0.002 | |
| PSM: propensity score matching  ^$^HR vaginal delivery comparator group; | | | | | | | | | | | | | |  |

| **Table G. Under-five mortality from external causes of death*, Brazil 2012-2018** | | | | | | |
| --- | --- | --- | --- | --- | --- | --- |
|  | **Deaths/1000 person years** | | **Propensity score matching** | | | |
| **Robson Groups** |  |  | **Before matching** | | **After matching** | |
|  | **Vaginal delivery** | **CD** | **HR (95% C)** | **p-value** | **HR (95% CI)** | **p-value** |
| **Robson1** | 0.14 | 0.09 | 0.69(0.61-0.79) | <0.001 | 1.04(0.89-1.21) | 0.613 |
| **Robson2a** | 0.12 | 0.09 | 0.78(0.62-0.99) | 0.040 | 1.04(0.77-1.40) | 0.805 |
| **Robson3** | 0.21 | 0.12 | 0.60(0.52-0.68) | <0.001 | 0.84(0.71-1.00) | 0.055 |
| **Robson 4a** | 0.16 | 0.13 | 0.83(0.63-1.09) | 0.182 | 1.06(0.72-1.54) | 0.764 |
| **Robson1-4 (2a, 4a)** | 0.17 | 0.10 | 0.62(0.57-0.67) | <0.001 | 0.95(0.86-1.06) | 0.387 |
|  |  |  |  |  |  |  |
| **Robson 5** | 0.20 | 0.13 | 0.66(0.58-0.74) | <0.001 | 0.89(0.76-1.05) | 0.172 |
|  |  |  |  |  |  |  |
| **Robson 6** | 0.27 | 0.12 | 0.42(0.25-0.70) | 0.001 | 1.08(0.55-2.12) | 0.814 |
| **Robson 7** | 0.32 | 0.17 | 0.53(0.38-0.74) | <0.001 | 0.86(0.56-1.33) | 0.499 |
| **Robson 8** | 0.40 | 0.22 | 0.54(0.42-0.70) | <0.001 | 1.05(0.76-1.45) | 0.745 |
| **Robson 9** | - | - | - |  | - |  |
| **Robson 10** | 0.32 | 0.20 | 0.63(0.57-0.71) | <0.001 | 0.90(0.79-1.02) | 0.101 |
| **Robson 6-10** | 0.32 | 0.19 | 0.58(0.53-0.63) | <0.001 | 0.90(0.81-1.02) | 0.105 |
| **General** | 0.19 | 0.14 | 0.71(0.68-0.74) | <0.001 | 1.00(0.94-1.08) | 0.865 |
| *ICD -10 Chapter XV: V01-V99 transport accidents, W00-W19 Falls, W20-W49 Exposure to inanimate mechanical forces,W50-W64 Exposure to animated mechanical forces,W65-W74 Accidental drowning and submersion,W75-W84 Other accidental hazards to breathing, W85-W99 Exposure to electric current, radiation and extreme ambient temperatures and pressures, X00-X09 Exposure to smoke, fire and flames,X10-X19 Contact with a heat source or hot substances, X20-X29 Contact with poisonous animals and plants, X30-X39 Exposure to the forces of nature, X40-X49 Accidental poisoning and exposure to harmful substances, X50-X57 Excessive efforts, travel and hardship, X58-X59 Accidental exposure to other and unspecified factors, X60-X84 Intentional self-harm, X85-Y09 Assaults | | | | | | |

CD- caesarean delivery

| **Table H. Hazard ratios from sensitivity analyses for under 5 mortality** | | | | | | | | |
| --- | --- | --- | --- | --- | --- | --- | --- | --- |
| **Robson Groups** | **Propensity score matched analysis (reference)** | | **Adjusted Cox model 3^*^** | | **Finer caliper^$^** | | **Propensity score with interaction terms** | |
|  | **HR (95% CI)** | **p-value** | **HR (95% CI)** | **p-value** | **HR (95% CI)** | **p-value** | **HR (95% CI)** | **p-value** |
| **Robson1-4 (2a, 4a)** | 1.25(1.22-1.28) | <0.001 | 1.21(1.18-1.24) | <0.001 | 1.28(1.25-1.32) | <0.001 | 1.25(1.22-1.29) | <0.001 |
|  |  |  |  |  |  |  |  |  |
| **Robson5** | 1.05(1.00-1.10) | 0.024 | 1.07(1.03-1.11) | <0.001 | 1.06(1.01-1.11) | 0.017 | 1.05 (1.00-1.10) | 0.045 |
| **Robson6-10** | 0.90(0.89-0.91) | <0.001 | 0.76(0.76-0.77) | <0.001 | 0.87(0.86-0.88) | <0.001 | 0.92(0.91-0.94) | <0.001 |
| ^*^adjusted by maternal age, education, marital status, baby sex, number prenatal care appointments, birth weight, year of birth, maternal race/ethnicity, IDH of the maternal municipality of residence and Robson group in combined analyses  ^$^ caliper of width 0·05 (in the primary analyses we used 0·1) | | | | | | | | |
