# Supplementary figures and images for: Associations between cesarean delivery and child mortality: A national record linkage longitudinal study of 17.8 million births in Brazil
Source: PLoS Med. 2021 Oct 12;18(10):e1003791. doi: 10.1371/journal.pmed.1003791 (PMC8509988; doi:10.1371/journal.pmed.1003791)

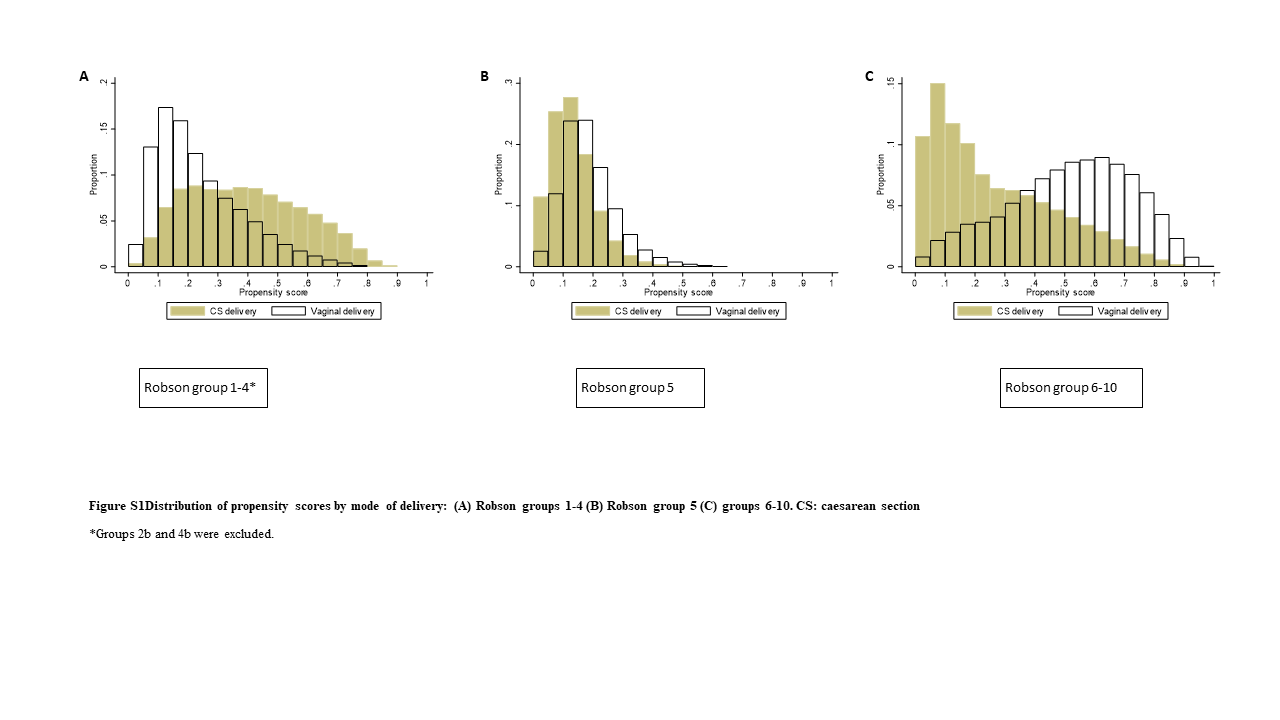

Supplement: S1 Fig — Distribution of propensity scores by mode of delivery: (A) Robson groups 1 to 4, (B) Robson group 5, and (C) Robson groups 6 to 10. * Groups 2b and 4b were excluded. CS, cesarean section. (TIF) [file pmed.1003791.s003.TIF]
