# Supplementary material for: Associations between cesarean delivery and child mortality: A national record linkage longitudinal study of 17.8 million births in Brazil
Source: PLoS Med. 2021 Oct 12;18(10):e1003791. doi: 10.1371/journal.pmed.1003791 (PMC8509988; doi:10.1371/journal.pmed.1003791)
Supplement: Attachment — Submitted filename: point by point R1 final.docx [file pmed.1003791.s004.docx]

**Manuscript reference number (PMEDICINE-D-21-00836R1)**

**Title: " Caesarean deliveries and child mortality: a national record linkage cohort study of 17.8 million births in Brazil "**

**Request from the editors**

| 1.Title: Please revise your title according to PLOS Medicine's style. Your title must be nondeclarative and not a question. It should begin with main concept if possible. "Effect of" should be used only if causality can be inferred, i.e., for an RCT. Please place the study design ("A randomized controlled trial," "A retrospective study," "A modelling study," etc.) in the subtitle (ie, after a colon). | We have replaced the title: *"Caesarean delivery and subsequent child mortality: a national record-linkage longitudinal study of 17.8 million births in Brazil”.* |
| --- | --- |
| 2. Data availability statement: Please provide a more direct link to the specific datasets used in the study, and, if possible, a contact email address for requests for data access. | The data availability statement was amended:  *All data supporting the findings presented here were obtained from Centro de Integração de Dados e Conhecimentos para Saúde (CIDACS). Importantly, restrictions apply to the availability of these data. However, upon reasonable request and provided all ethical and legal requirements are met, the corresponding author will make the data available. Information on how to apply to access the data can be found at https://cidacs.bahia.fiocruz.br/.* |
| 3. Abstract: Methods and Findings: Please quantify all main results presented in the abstract with both 95% CIs and p values. | We have added the p value to the 95%CIs in the abstract |
| 4. Abstract: Methods and Findings: In the last sentence of the Abstract Methods and Findings section, please describe the main limitation(s) of the study's methodology. | We have included this sentence: *Our results should be interpreted with caution in clinical practice, since relevant clinical data on CD indication were not available.* |
| 5. Abstract: Conclusions: Please address the study implications without overreaching what can be concluded from the data; the phrase "In this study, we observed ..." may be useful. | We have included this sentence: *In this study we observed that in Robson groups with low expected rates of CD, this procedure was associated with a 25% increase in child mortality. However, in groups with high expected rates of CD, the benefits of clinically-indicated CD were confirmed.* |
| 6. Author summary: At this stage, we ask that you include a short, non-technical Author Summary of your research to make findings accessible to a wide audience that includes both scientists and non-scientists. The Author Summary should immediately follow the Abstract in your revised manuscript. This text is subject to editorial change and should be distinct from the scientific abstract. Please see our author guidelines for more information: [https://journals.plos.org/plosmedicine/s/revising-your-manuscript#loc-author-summary](https://protect-eu.mimecast.com/s/UwafCX5WOI4N8p1iMIMCH?domain=journals.plos.org) | We have now included the Author summary in the manuscript. |
| 7. Throughout the text: Please place in-text citations within square brackets, before the punctuation, like this [1]. When multiple references are included, please do not include spaces within the brackets. | Done |
| 8. Introduction: Lines 84-85: Please revise this sentence to avoid causal implications: “... we investigated the effect of CD on child mortality…” | Thank you for pointing this out. We have replaced the objective: *we investigated the association between CD and child mortality according to the Robson classification and explored the relationship between pre- and post-labour CD, and risk of death in offspring*. |
| 9. Methods: Please ensure that the study is reported according to the STROBE guideline, and include the completed STROBE checklist as Supporting Information. Please add the following statement, or similar, to the Methods: "This study is reported as per the Strengthening the Reporting of Observational Studies in Epidemiology (STROBE) guideline (S1 Checklist)." | We included the following statement and included the complete checklist as supplementary material S1: *This study is reported as per the Reporting of studies Conducted using Observational Routinely-collected Data (RECORD) guideline (S1 Checklist).* |
| 10. Methods: Did your study have a prospective protocol or analysis plan? Please state this (either way) early in the Methods section.  a) If a prospective analysis plan (from your funding proposal, IRB or other ethics committee submission, study protocol, or other planning document written before analyzing the data) was used in designing the study, please include the relevant prospectively written document with your revised manuscript as a Supporting Information file to be published alongside your study, and cite it in the Methods section. A legend for this file should be included at the end of your manuscript.  b) If no such document exists, please make sure that the Methods section transparently describes when analyses were planned, and when/why any data-driven changes to analyses took place.  c) In either case, changes in the analysis-- including those made in response to peer review comments-- should be identified as such in the Methods section of the paper, with rationale. | *We did not have a written analysis plan; however, we specified the list of confounders and categorisations of the continuous variables before analysing the data.* |
| 11. Results: Line 173: Please define the abbreviation “PSM” at first use, and use consistently after. | We have included the abbreviation PSM in the methods section, line 175 and used consistently after. |
| 12. Results: Please provide p values in addition to 95% CIs for all results presented in the text. | We have included the p values in the results section of the paper, and all other sections. |
| 13. Discussion: Line 270 and 271-272: Please revise these sentences to avoid causal implications: “We confirmed the protective effect of CD on non-cephalic presentation.” and “Moreover, our study showed a strong protective effect of CD among pregnancies with multiple and preterm births.” | We have now substituted words to avoid causal implications. *We confirmed the protective association of CD on non-cephalic presentation, similar to those benefits seen in observational studies and clinical trials [25,26]. Moreover, our study showed a strong protective association of CD among pregnancies with multiple or preterm births.* |
| 14. Discussion: Line 300: Please include the missing reference. | Thank you. We have included the missed reference. |
| 15. Discussion: Line 304-305: Please revise this sentence to avoid implying causality: “In summary, our results provide evidence that either overuse or underuse of CD can affect child survival adversely.” | We have now substituted the words to avoid causal implications: *In summary, our results provide evidence that either overuse or underuse of CD is associated with child survival.* |
| 16. References: Please use the "Vancouver" style for reference formatting, and see our website for other reference guidelines [https://journals.plos.org/plosmedicine/s/submission-guidelines#loc-references](https://protect-eu.mimecast.com/s/oNBgCZ01Qi7RkOEcJZ5T9?domain=journals.plos.org) | We have reformatted references using the Vancouver style. |
| 17. Table 1: Please define abbreviations “HDI” and “CD” in the legend. Please confirm if CS should be CD (as used elsewhere in the manuscript). Please include in the legend that values are number and percent. | Done |
| 18. Table 2: Please include p values for both the unmatched and propensity score matched results. In the left hand column, please indicate these are Robson groups. | Done |
| 19. Table 3: Please define the abbreviation “CD” in the legend, and please indicate the left hand column represents the Robson group. Please also provide the p values in addition to the 95% CIs for the adjusted and unadjusted analyses. | Done |
| 20. Figure 1: We suggest a more descriptive title for this figure would be helpful. | We have amended the title of figure 1*: Flow chart of cohort selection process and the Robson classification.* |
| 21. Figure 2: Please include a legend describing the six panels, A-F, and we suggest a more descriptive title. | Done. New title: *Figure 2: Kaplan-Meier curves of mortality from birth up to five years of age for vaginal and caesarean births by Robson group* |
| 22. Table S1: Please define the abbreviation “CS” | We replaced for caesarean delivery (CD). |
| 23. Table S2, S3, and S4: Please define the abbreviation “HDI” and “CS” in the legend. | Done |
| 24. Table S5: Please provide p values in addition to 95% CIs. Please define “HDI” and “CS” in the legend. | Done |
| 25. Table S6: Please include p values in addition to 95% CIs. Please indicate in the legend that “before” and “after” refer to propensity score matching. | Done |
| 26. Table S7: Please include p values in addition to 95% CIs. | Done |
| 27. Table S8: Please revise the title to avoid causal language. Please include p values in addition to 95% CIs. Please define the abbreviation “PSM” in the legend. | Done |

Reviewer #1:

| It might be useful to give deaths per capita (or per 100,000 babies or something) in addition to per person year. Person-year is appropriate statistically, but not very intuitive. Doctors and patients probably want to know "What is the risk that the baby dies?" One way to address this is to do logistic regression in addition to Cox. However, a rough estimate of this can (if I am thinking straight) be gotten by dividing the person-year figure by about 5. | Thank you for your suggestion. We have included the mortality risk (deaths per 1000 live births) from birth up to 5 years of age by mode of delivery in Table 2. |
| --- | --- |
| p. 2 line 41-2 Insert "significant" There was some evidence | Done |
| Table 1 I would give the percentage row-wise rather than column-wise. I am more interested in, e.g. what proportion of group 1 were vaginal vs. CD rather than what proportion of vaginal births were group 1 | Done |
| Table 2: Not surprisingly, nearly all the deaths were in the risky groups. I think more could be said about this. For a doctor and patient trying to decide whether to have vaginal delivery or CD, the key factor is not the proportion of added risk in odds ratio or hazard ratio terms, but the actual risk of the baby dying. Going from a risk of 1 in 2000 to a risk of 1 in 1800 is not the same as going from 1 in 20 to 1 in 18. Some of this can be seen in fig 2, | Thank you for point this out. We have added mortality risk information in table 2. and described this in the text.  The mortality risk varied significantly by Robson groups as well as by the mode of delivery. Robson groups 6-10 had the highest mortality risks. Robson group 6 had the highest mortality risk among those delivered vaginally and largest difference with those delivered via caesarean, 131.5 per1000 live births vs 2.0 per1000 live births. This was followed by group 8 which was, 91.1 per 1000 live births vs 24.1 per 1000 live births respectively. The mortality risk differences between vaginal and caesarean delivery was not so marked in groups 1-5 (Table 2). |
| Non-statistical point of interest: What about women who had CD for a reason related to their own health? For instance, my ex-wife had CD because she has a shunt in her belly and the ob-gyn thought that labor might induce gastrointestinal fluid to go into her brain. I have no idea how common such things are, but if they are at least somewhat common, they might be worth a comment - they don't seem to be captured in Robson. | Yes, you are right. We cannot address this using the Robson classification. Because of this we included the lack of such relevant individual clinical level data as the main limitation of our study (a lack of data about the caesarean delivery indication). However, the fact that the increased mortality risk following CD compared with vaginal birth but not with unscheduled (emergency) CD compared to pre-labour CD suggests that confounding by indication for the CD(the issue you raise) is not the explanation for the association that we found. |

Reviewer #2

| 1) there is no hypothesis presented, which is tested in this study. Please add a clear hypothesis. | Thank you. We have included a clear hypothesis. *We hypothesised that in groups with a low indication for CD, this procedure would be associated with higher child mortality. On the other hand, in groups with clear medical indication, we hypothesised that CD is likely to improve child survival chances.* |
| --- | --- |
| 2) The sample is not described sufficiently. Please indicate inclusion and exclusion criterions | We have included the following information: *Once data were linked, all records were eligible to be part of the study, we only excluded records with contradictory data (e.g. simultaneously reporting no previous pregnancy and a previous vaginal delivery) or records with incomplete information on the mode of delivery, previous pregnancy, gestational weeks at delivery, number of fetuses, delivery onset or a previous CD.* |
| 3) Please add a table containing the sample characteristics. maternal age, sociodemographioc characteristics, reproductive history, independent of Robson criterions. Just for the whole sample. | Done, we have included Table S3. |

Reviewer #3:

| would be interesting to examine maternal mortality/near miss morbidity | Yes, we are analysing the maternal mortality and it will be a follow up paper. |
| --- | --- |
| could be followed by a paper on how many lives saved modelling these findings to other national or regional data | Thank you for this brilliant idea. It sounds interesting, we will consider a paper modelling these results. |
| - line 177 scoire - line 194; should be if instead of it -line 301 at risk.Or | We have corrected these typos. Thank you. |
