# Supplementary material for: Associations between cesarean delivery and child mortality: A national record linkage longitudinal study of 17.8 million births in Brazil
Source: PLoS Med. 2021 Oct 12;18(10):e1003791. doi: 10.1371/journal.pmed.1003791 (PMC8509988; doi:10.1371/journal.pmed.1003791)
Supplement: Attachment — Submitted filename: point by point R2 ok.docx [file pmed.1003791.s005.docx]

**Manuscript reference number (PMEDICINE-D-21-00836R1)**

**Title: "Associations between caesarean delivery and child mortality: A national record-linkage longitudinal study of 17.8 million births in Brazil "**

**Request from the editors**

| 1. Title: Please capitalize the first word of the subtitle, and we suggest revising to: “Associations between caesarean delivery and subsequent child mortality: A national record-linkage longitudinal study of 17.8 million births in Brazil” or similar. | Done |
| --- | --- |
| 2. Abstract: Background: Please clearly state the study hypothesis as the final sentence. | We have added the sentence. **New text**: Our hypothesis was that in groups with a low indication for CD, this procedure would be associated with higher child mortality and in groups with a clear medical indication CD would be associated with improved child survival chances. |
| 3. Abstract: Methods and findings: Please check the p value reported for this finding: “For the live births to women with a CD in a prior pregnancy (Robson group 5), the relative rates for child mortality were similar for those born by CD compared with vaginal deliveries (HR=1.05, 95% CI:1.00-1.10; p=0.024).” | Done. It is correct. |
| 4. Abstract: Conclusion: We suggest replacing “the benefits of clinically-indicated CD were confirmed.” with “...we observed a reduction in child mortality.” As Robson group was used as a proxy for CD indications, we suggest tempering the language here: “These findings may suggest that clinically-indicated CD is associated with child outcomes.” or similar. | We have replaced this sentence. **New text**: However, in groups with high expected frequencies of CD, the findings suggest that clinically-indicated CD is associated with a reduction in child mortality. |
| 5. Author summary: Why was this study done?: Please revise the third point to “Unindicated CDs add to costs, potentially introduce harms, and the effects of unindicated CD on infant and child health outcomes remain unclear, especially in low and middle income countries.” | Done as suggested. |
| 6. Author summary: Please use the abbreviation "CD" throughout after it is first introduced. | Done as suggested |
| 7. Author summary: What did the researchers do and find?: Please combine the first two bullet points. Please clarify the third bullet point to make it clear if this is among women with a previous CD, or among all live births. | We clarified to make it clear that we are presenting the results for Robson group 1-4. |
| 8. Author summary: What do these findings mean? Please revise the first bullet point to remove causal implications: “Our study suggests that, in Brazil, CD can be associated with increased risk of child mortality unless there is a clear indication for the procedure.” | Done as suggested |
| 9. Methods: Page 7-8: Please identify the confounders included in the propensity score model and adjusted for in the Cox proportional hazards model, and also include the information on how each was reported/assessed and categorized for the analysis. | We included the variables used as confounders. **New Text**: The propensity score model matched on potential confounders available in the SINASC dataset: maternal age (in 2-year groups); level of maternal education (none, 1-3; 4-7, 8-12 and more than 12 years of schooling); self-declared maternal race/ethnicity (White, Black, Asian, Mixed race, Indigenous); marital status (single, widow, divorced, married/union); HDI of the municipality of residence of the mother (≥0.80, <0.80 to 0.70, <0.70 to 55, <0.55); number of prenatal care appointments (none, 1-3, 4-6, more than 7 appointments); sex of newborn (female or male); birth weight (in 200g categories); year of birth and Robson group (1-10). Interaction terms were not included in the models. To assess the robustness of the propensity score matching, we also conducted an alternative analysis using a conventional Cox proportional hazards model adjusted for the same confounders used to estimate the propensity score. |
| 10. Methods: Please clarify how statistical significance of differences between comparison groups was determined. | We added this sentence. **New Text:** We used Kaplan-Meier estimates of mortality risk and Cox regression to compare mortality in the first five years of life by caesarean versus vaginal mode of delivery using the age of the child in days since birth for the time scale in our survival analyses. Statistical significance was defined as 95% confidence intervals that excluded 1.0, with *p*-values calculated accordingly |
| 11. Results: “The mortality risks between vaginal and caesarean deliveries were not so markedly different in groups 1-5 (Table 2).” Please make it clear in the text if these differences were statistically significant or not. | Thank you. We amended the sentence. **New text:** The mortality risks between vaginal and caesarean deliveries were not statistically significant different in groups 1-4. |
| 12. Results: “In a breakdown by time-period, unscheduled CD was associated with increased rate during the neonatal period (HR=1.18, 95% CI: 1.05-1.32; p=0.003) in group 2 and in the first year of life (HR=1.15, 95% CI: 1.03-1.29; p=0.010) in group 4 (Table 3).” Please clarify if “associated with an increased mortality rate during the neonatal period…” is meant. | Done. **New text:** In a breakdown by time-period, unscheduled CD was associated with an increased mortality rate during the neonatal period (HR=1.18, 95% CI: 1.05-1.32; p=0.003) in group 2 and in the first year of life (HR=1.15, 95% CI: 1.03-1.29; p=0.010) in group 4 (Table 3). |
| 13. Discussion: First sentence: We suggest beginning with “In a propensity score-matched analysis, we observed that among live births to women with low expected CD rates (Robson group 1-4), who had a CD…” | Done. |
| 15. Discussion: “We conducted the sensitivity analysis to quantify residual confounding due to socioeconomic differences between women delivered vaginally and via caesarean section and confirmed there was little.” Please revise this sentence, as the findings of the sensitivity analysis do not confirm that there was little residual confounding. Rather, the sensitivity analysis did not find evidence suggestive of residual confounding, but that does not confirm that residual confounding may not be present. | Thank you for pointing this out. We have amended the sentence accordingly. **New text:** We conducted the sensitivity analysis to quantify residual confounding due to socioeconomic differences between women delivered vaginally and via caesarean section and we did not find evidence suggestive of residual confounding. |
| 16. Conclusion: “We recommend further research in low- and middle-income settings to confirm our finding that CD increases the risk of child mortality…” Please revise this sentence to avoid causal implications. | We amended accordingly. **New text**: We recommend further research on non-medical indicated caesarean section in low- and middle-income settings to confirm our finding that CD is associated with an increased risk of child mortality and to explore the effects of CD on child morbidity. |
| 17. Competing interests, Financial Disclosures and Data Availability: Please remove these sections from the main text of the manuscript. This information will be taken directly from the relevant sections of the manuscript submission metadata. | Done |
| 18. References: Please double check each reference for the correct formatting. Please use the correct abbreviations for journal titles (e.g. “Lancet” vs “The Lancet”). Please use the "Vancouver" style for reference formatting, and see our website for other reference guidelines [https://journals.plos.org/plosmedicine/s/submission-guidelines#loc-references](https://protect-eu.mimecast.com/s/1V1JC9gWMIRQmk6SZgc1N?domain=journals.plos.org) | Done |
| 19. Figure 2 and Figure S1: Please provide descriptive legends for each of these figures. | Done |
| 20. Supporting information Table S3: Please include this table in the main text of the paper, rather than as a supporting information table. | Done |
| 21. Table S10: Please clarify in the legend whether the “primary analysis” represents the Propensity Score Matched analysis. | Done |
| 22. We suggest including the RECORD checklist as a separate file. | Done |
|  |  |

Reviewer #1:

| The authors have addressed my concerns and I now recommend publication | Thank you. |
| --- | --- |

Reviewer #2

| In my opinion, the authors have addressed all suggestions of the reviewers. Therefore, I recommend to accept this manuscript in its present form. | Thank you*.* |
| --- | --- |
